# Application of Recombined Milk to Produce Crescenza-Type Cheese in Laboratory-Scale Cheesemaking: Implications on Technology and Sensory Properties

**DOI:** 10.3390/foods9070928

**Published:** 2020-07-14

**Authors:** Flavio Tidona, Salvatore Francolino, Roberta Ghiglietti, Francesco Locci, Gianluca Brusa, Marcello Alinovi, Germano Mucchetti, Giorgio Giraffa

**Affiliations:** 1 CREA-ZA, Research Centre for Animal Production and Aquaculture, 26900 Lodi, Italy; flavio.tidona@crea.gov.it (F.T.); salvatore.francolino@crea.gov.it (S.F.); roberta.ghiglietti@crea.gov.it (R.G.); francesco.locci@crea.gov.it (F.L.); gianluca.brusa@crea.gov.it (G.B.); 2 Food and Drug Department, University of Parma, 43100 Parma, Italy; marcello.alinovi@studenti.unipr.it (M.A.); germano.mucchetti@unipr.it (G.M.)

**Keywords:** skimmed milk powder, recombined milk, Crescenza-type cheese, mini-cheesemaking, cheese processing

## Abstract

This work evaluated the effect of recombined skimmed milk (RM), mixed in different ratios (40, 60, and 100%) with fresh cow milk, on the processing technology and quality of Crescenza, an industrial soft cheese of the Italian dairy tradition. Crescenza-type cheeses were produced at a laboratory scale, following the industrial process. Control cheese consisted of Crescenza-type cheese produced with 100% whole fresh milk. Compared to control cheese, the substitution of fresh milk with 60–100% of RM deteriorated the coagulation properties and led to a higher moisture retention, whereas, with 40% of RM, the differences were not statistically significant. Cheeses produced with any concentration of RM, although of acceptable quality, differed significantly in terms of sensory properties from control cheese. The addition of colloidal calcium phosphate, or CaCl_2_ together with a reduction in the size of the curd at cutting, minimized the differences in composition and sensory properties between cheeses produced with 40% RM and control cheese. This study suggested the applicability of 40% RM to obtain Crescenza-type cheese with suitable quality characteristics. The type of product, the technology, the quality, and quantity of the powders are all key factors to be taken into account for a successful application.

## 1. Introduction

In some countries, i.e., USA, New Zealand, and Germany, the production of milk in larger amounts than national demand and the development of a more energy-efficient milk powder manufacturing process [1] favored the availability of high stocks of milk powder (MP), which can be easily transported and stably stored over time [2]. MP can be used for milk standardization as an alternative to phosphocasein (PC) or milk protein concentrates (MPC), but it can also be employed to produce cheeses in many developing countries, where the availability of fresh milk is scarce or affected by seasonal shortage [3,4]. The need to use MP in cheesemaking may also be related to economic reasons, depending on the fluctuation of milk market prices, which may ultimately affect a company’s productivity and competitiveness. The production of different types of cheeses from recombined milk (RM), such as Ras, Domiatti, Cheddar, Halloumi, and Mozzarella, [4,5,6,7] has been reported. To manufacture RM products at a large scale, technical skills and advanced equipment have been implemented by the main dairy exporting countries (New Zealand, Australia, and Denmark), although efforts to build local dairy processing industries were also made in South East Asia, Latin America, Africa, and the Middle East [8]. Studies on Cheddar, Cottage, and Quark indicate that the quality of the starting powder decisively influences the quality of the cheeses obtained. In particular, the heating conditions throughout the powder manufacturing process may determine the denaturation of whey proteins and their interaction with other proteins, influencing the chemical and physical properties of the derived cheeses [2,9,10]. An important issue is also the amount of MP that can be used, as it can be possible to obtain Mozzarella cheese with good meltability and stretchability using a 40–60% of RM [10], whereas higher amounts lead to cheeses with higher moisture content and harder curd [11]. Cheeses from RM have never been produced in Italy, as the Italian Law N. 138 of 11 April 1974 forbids the storage and use of powdered, condensed, or reconstituted milk in cheese production.

In this work, a laboratory-scale cheesemaking system was developed to produce Crescenza-type cheeses with RM. The production of Crescenza, an Italian soft cheese with a short maturation, accounts for 28,000 tons per year and currently represents a market share of 30% compared to the total of Italian, non-PDO soft cheeses, with a rising trend of consumption in the domestic market [12]. The effects of the use of different concentrations of RM in place of fresh milk in the manufacture of Crescenza-type cheese, with focus on the impact on the processing and quality of the products thereof, were studied. Cheeses obtained with RM were compared with control cheese, e.g., Crescenza-type cheese made with 100% of fresh milk.

## 2. Materials and Methods

### 2.1. Evaluation of Renneting Properties of the Milk Powder (MP)

Skimmed MP (batch n. 3/215) was supplied by S.C.A. Srl (Fiorenzuola d’Arda, PC, Italy). Whey protein nitrogen index (WPNI), which is a well-known method for classifying milk powders in relation to the intensity of heat treatments applied during their preparation, [13] was determined according to Association of Official Analytical Chemists (AOAC, 1980) [14]. A preliminary evaluation of the renneting properties of MP was performed by the Formagraph instrument (Foss Electric, Hillerød, Denmark) using raw and pasteurized (73 °C for 20 s) fresh milk as the control (control milk). Fresh milk was provided by the CREA-ZA farm of Lodi, and its composition was determined by infrared spectroscopy using a MilkoScan FT2 spectrophotometer (Foss, Padova, Italy). MP was reconstituted at the same protein (approx. 3.40) and fat (3.90) percent of the control milk. The following parameters were measured at 37 °C: clotting time (r) and firming time (K20), which were calculated from the addition of rennet (0.088 IMCU/mL milk; Naturen^®^ Extra, Chr. Hansen, Denmark), and curd firmness after 30 min (A30) [15]. The effect of two technological aids, i.e., CaCl_2_ at 0.1 g/L and a dispersion of colloidal calcium phosphate (CPC) (Calimix CAL, S.C.A. Srl, Fiorenzuola, Italy) at 1 mL/L, was also evaluated. Averages and standard deviation of five measurements were calculated.

### 2.2. Preparation of Recombined Milk (RM)

Skimmed MP was reconstituted (10 ± 0.27 g/100 g) with distilled H_2_O at 24 °C and dissolved for 30 min with a magnetic stirrer for complete hydration, until the protein concentration was equivalent to that of control milk. Pasteurized, not homogenized, milk cream with 37% fat (COOP, Casalecchio di Reno, BO, Italy) was then added to standardize the fat content of the reconstituted skimmed MP to 3.9% fat content, and the mixture was kept overnight at 4 °C under gentle stirring to obtain RM. Different blends (RM: fresh milk), i.e., 40:60, 60:40, and 100:0 % (*w*/*w*), were studied.

### 2.3. Set Up of a Laboratory Cheesemaking System

**Figure 1:**
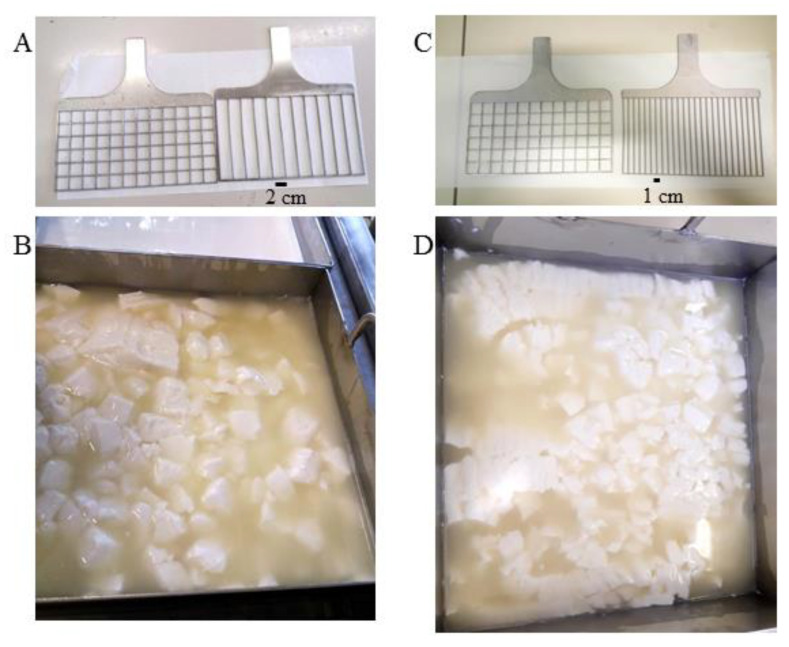
Cutters used for all the lab-cheesemaking productions (**A**) with the relative curd obtained after cutting (**B**) and the modified cutters (**C**) used to reduce the coagulum size after cutting (**D**).

**Figure 2:**
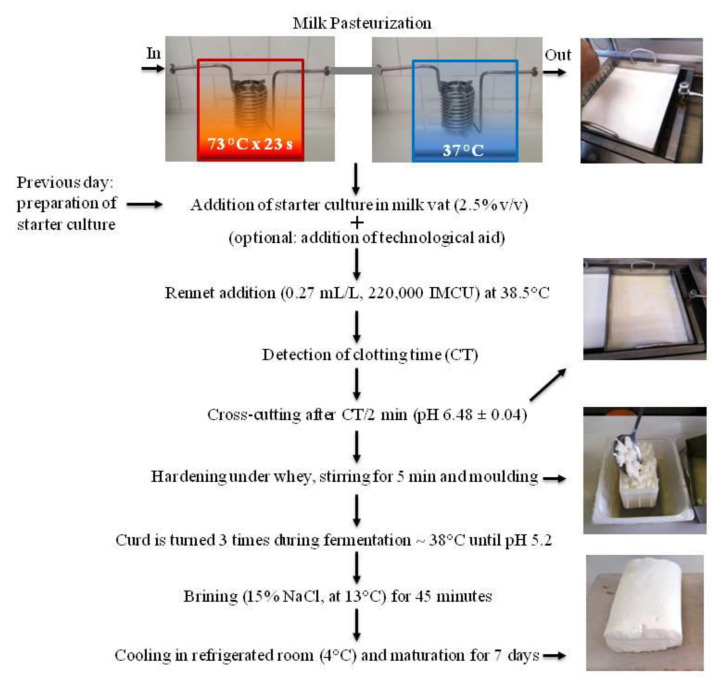
Flowchart of the lab-cheesemaking process set up for this study to produce Crescenza-type cheeses.

Small-scale Crescenza-type cheeses were manufactured in 10 L in vats following the industrial process. Milk (both fresh and RM: fresh blends) was pasteurized with a self-assembled tubular heat exchanger, specifically designed to obtain a continuous flow treatment. The heat exchanger was composed of three water baths set at different temperatures, the first acting as a preheating section, the second as a holding section, and the third for milk cooling. Continuous steel tubes with an internal diameter of 6 mm and with a different length, which had been calculated to obtain the required temperature profile, were lodged into the baths and connected in series using tri-clamp connections. The milk’s flow rate in the tubular exchanger was set at 19 L/h by means of a peristaltic pump (Masterflex Easy-Load 7518-10, ALT, east Lyme, Connecticut, USA) equipped with an inverter; a pre heating section of 3.2 m was applied to reach the set temperature of 73 °C and a holding section of 6 m corresponding to a time of 16 s for the fastest particle was used to set the duration of the heat treatment. The cooling section, 10 m in length, was set at 37 °C to approach the coagulation temperature of 38.5 °C of milk in vat. The day before production, a starter culture consisting of *Streptococcus thermophilus* was prepared. For this purpose, a freeze-dried commercial culture (ST022, Sacco Srl, Cadorago, CO, Italy) was inoculated in reconstituted skimmed MP (sterilized at 110 °C for 30 min), incubated at 38.5 °C, until reaching pH~5, and stored at 4 °C. The culture was added to a milk vat at an inoculum level of 2.5 ± 0.2 % (*w*/*w*), followed by rennet (Naturen^®^ Extra, 220 IMCU/mL, Chr. Hansen, Denmark), which was added at a concentration of 0.27 mL/L to the vat milk set at 38.5° C. Starting from the addition of rennet, the time to reach milk gelation (clotting time (CT)) was measured. The curd was then cut after CT/2 min, corresponding to half of the clotting time detected in each trial, to reach a similar gel firmness at cutting across the trials. The curd was cross cut at pH 6.48 ± 0.04 in cubes sized 2 × 2 × 2 cm^3^ (Figure 1A), left under whey to allow gel firming, and gently stirred (Figure 1). The curd was then discarded from the vat into a mold with a square-shaped base and kept at 38 °C in a conditioned chamber (Thermostatic Dubnoff bath, International PBI, Milan, Italy) until a pH value of 5.2 was reached. Temperature and pH were recorded by portable pHmeters (Portavo 907, Knick, Beuckestr, Berlin, Germany). After molding and during the turning of the curd, the whey released was carefully collected, weighed, and analyzed by FT-IR (see below). The cheeses were then salted by immersion in brine (NaCl 15% *w*/*w*, 13 °C) for 45 min and then transferred to a refrigerated room (4 °C) for 7 days to complete the drainage of the whey and the salt diffusion. Cheeses were then ready for sale. The shelf life of the Crescenza was about 20 days. The flowchart of the cheesemaking process is summarized in Figure 2. Cheeses made with different blends of RM, such as fresh milk and control cheese (made with 100% fresh milk), were produced on the same day, and all the trials were replicated three times. To assess whether they fell within the quality range of commercial cheeses available on the market, control cheese was first compared with four commercial Crescenza cheeses (named cheese-1, cheese-2, cheese-3, and cheese-4), produced by some of the market leaders in Italy.

### 2.4. Crescenza-Type Cheeses with 40% RM and Technology Optimization

Two further lab-cheesemakings at the lowest concentration (40%) of RM were carried out in separate experiments and compared to control cheese. In the first trial, a dispersion of CPC (Calimix CAL, S.C.A. srl, Fiorenzuola, Italy) at 0.5 mL/L was added to RM; in the second trial, CaCl_2_ at 0.05 g/L was added to RM, followed by a change in a cutter (Figure 1C), which gave rise to smaller (2 × 2 × 1 cm^3^) curd grains (Figure 1D). Trials were replicated three times.

### 2.5. Cheese Composition and Yield

Fat, protein, lactose, dry matter content of milk, and drained whey were determined by FT-IR, using a MilkoScan FT2 (Foss, Hillerød, Denmark) soon after processing. The composition and cheese yield were analyzed at the beginning of the shelf life, i.e., the seventh day of age. Fat and protein were determined by MilkoScan; the dry matter was determined by weighing the residue after oven drying at 102 °C [16] and the total ash was determined gravimetrically after dry ashing at 550 °C (FIL-IDF 27, 1964). Sugars (lactose and galactose) and lactic acid were determined by HPLC, according to Bouzas et al. (1991) [17]. The same determinations were carried out on Crescenza cheeses from retail. Milk, whey, curd, and cheese masses were weighted with a precision scale of ± 0.1 g (IND 690 model; Mettler Toledo, Greifensee, Switzerland). Actual cheese yield (Ya), evaluated at the beginning of shelf life, was calculated by dividing the weight of cheese by the weight of milk × 100. Taking into account the estimated reference values for milk composition (fat 3.90%, proteins 3.45%) and cheese moisture content (58%), the corrected yield Ymafpam (moisture-adjusted, milk protein plus fat adjusted cheese yield) was expressed as previously reported by Alinovi et al. (2018) [18]. Analyses were carried out in duplicate.

### 2.6. Sensory Analysis

A quantitative descriptive analysis (QDA) of control and retail cheeses was performed to measure the product’s sensory characteristics, selecting five sensory descriptors (sourness, texture, taste, bitterness, and overall appreciation) to define the product profile. An ordinal sensory scale of intensity from 1 (low) to 9 (high) was applied. QDA was carried out at the mid-term of shelf-life for Crescenza (approx. 10 d), purchasing cheeses from retail with a residual 10–12 days from the expiry date, thus assuming similar manufacture periods between cheeses. The test was carried out by 40 skilled consumers.

A discrimination triangle test was performed to evaluate whether perceivable differences could be detected between cheeses made with RM and control cheese. To prevent any biased judgement, a randomized three-digit code (AAB, ABA, BAA, BBA, BAB, and ABB) was used to identify the samples administered to panel members (n between 20–30 people, aged 25–60 years old).

### 2.7. Statistical Analysis

The comparison among different samples was carried out by one-way analysis of variance (ANOVA) (α = 0.05) (Paleontological statistics software, PAST, Natural History Museum, Oslo, Norway). The significance of the triangle test was set at a α-level of 0.05, and the number of corrected responses was based on the critical values table reported by Lawless and Heymann (1998) [19].

## 3. Results

### 3.1. Validation of the Lab-Cheesemaking System

**Figure 3:**
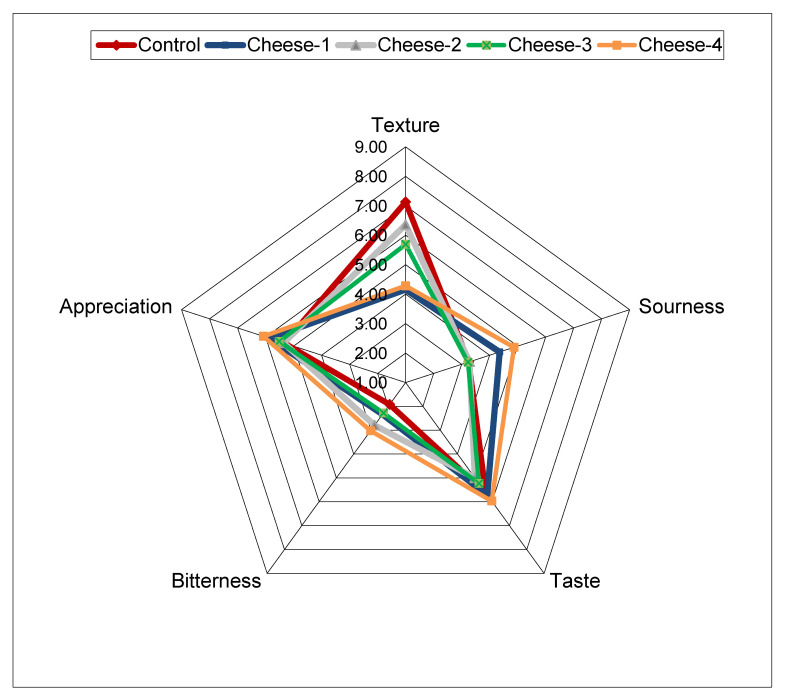
Spider plot from the quantitative descriptive analysis (QDA) carried out between lab scale control cheese and four commercial Crescenza cheeses from retail.

To evaluate the suitability of the lab-cheesemaking system (Figure 2), control cheese (produced with 100% fresh milk) was compared to four cheeses taken from retail. The chemical composition of all the cheeses resulted as comparable (Table 1) and in agreement with Crescenza product standards [20]. From the QDA analysis, panelists reported a harder texture in control cheese and cheese-2 compared to the others (Figure 3). Moreover, cheese-4 was perceived as significantly sourer by the panel. However, these minor differences fall within the commercial variability found in retailed Crescenza cheese. Overall, control and commercial cheeses were equally appreciated (Figure 3).

### 3.2. Evaluation of the Renneting Properties of RM

The WPNI of the MP used for this study was 5.25 ± 0.20 mg/g, which led to classify it as a medium heat MP. This value is very close to a low heat MP, ranked as such when the WPNI is ≥ 6 mg/g [21]. The evaluation of the renneting properties (r, K20, A30) showed significant differences (*p* < 0.05) between fresh milk (raw or pasteurized) and RM reconstituted at the same protein and fat content of fresh milk. RM displayed a significant delay in the formation of the coagulum and a weaker gel than both raw and pasteurized fresh milk. Notably, pasteurization did not significantly impact on coagulation parameters compared to raw milk (Table 2). The addition of CaCl_2_ or CPC improved some of the renneting properties of RM. The addition of CaCl_2_ or CPC allowed to obtain r values similar to or even lower than those observed with raw or pasteurized milk. Furthermore, the consistency (A30) of the RM coagulum was not improved with the addition of CaCl_2_ or CPC, while, with the use of the latter, a significantly shorter K20 was observed (Table 2). From these data, it was decided to halve the concentration of both CPC (from 1.0 to 0.5 mL/L) and CaCl_2_ (from 0.1 to 0.05 g/L) in the cheesemaking trials.

### 3.3. Effect of Different Blends of RM: Fresh Milk on the Cheese-Making Process and Cheese Composition

A significantly delayed clotting time with respect to control cheese was detected in cheeses produced with 100% and 60% of RM, whereas with 40% of RM, clotting time was slightly faster than control cheese, but the difference was not significant (Table 3). No significant differences in fermentation time, i.e., the time from the addition of the starter until the curd reached pH 5.2, were observed. Cheeses produced with 60 and 100% of RM showed a higher moisture content and a lower fat content than control cheese (Table 3). The higher moisture content of 100% RM cheese contributed to a significantly higher cheese yield (Ya). However, the corrected cheese yield (Ymafpam) did not show significant differences between control and RM cheeses, regardless of the % MP replacement (Table 3). Despite some differences, all the milk blends appeared suitable to be used in cheesemaking and provided cheeses with composition and characteristics within the standard range for Crescenza [20]. Since cheeses produced with 40% of RM were the most comparable with cheeses made with 100% fresh milk, they were chosen for further tests.

### 3.4. Crescenza-Type Cheeses with 40% RM and Technology Adaptation

The aim of this part of the study was to obtain RM cheeses with a moisture content similar to the control cheese. Compared to the control cheese, the use of both technological aids (0.5 mL/L CPC or 0.05 g/L CaCl_2_) to produce Crescenza-type cheeses with 40% RM enabled us to obtain similar clotting times, avoiding the small delay previously observed using only RM, and a comparable whey drainage rate during manufacturing (Table 4). The overall volume of the whey collected at the end of the fermentation was similar between CaCl_2_, CPC, and control cheese. Moreover, the addition of CaCl_2_ coupled with the reduction of the curd grains size carried out by a modified cutting tool (Figure 1C,D) allowed the final composition of the cheeses obtained with 40% RM to be similar to control cheese. A slightly, but statistically not significant (*p* > 0.05), higher fat content in the whey, released by the 40% RM cheeses compared to the control cheese, was observed (Table 5). The weaker structure of the coagulum observed with 40% RM at the cutting time may lead to the loss of more fat globules in the whey. Lactose in drained whey and lactose, galactose, and lactic acid in Crescenza-type cheeses after 7 days of maturation did not significantly differ between control and experimental cheeses (Table 5), thus showing that the use of RM did not interfere with the lactic fermentation. The Ya was very similar in all cheeses and coherent with yields expected from Crescenza cheese [18]. Because of the similar cheese moisture content, the corrected yield Ymafpam of RM cheeses was expectedly similar to control cheese (Table 4).

### 3.5. Sensory Properties

To detect possible sensorial differences between cheeses manufactured with RM and fresh milk, a triangle test was applied. Significant differences from control cheese were perceived in cheeses made with 100, 60, and 40% RM (Table 6). Slight optimizations of the production process in cheeses made with 40% RM (addition of CaCl_2_ combined with a reduction in the curd grain size) enabled us to obtain cheeses very similar to the control cheese (Table 6).

## 4. Discussion

The use of MP or other anhydrous milk derivatives (e.g., milk protein concentrates or caseinates) is a well-established practice in dairy technology as it has the advantage, among others, of obtaining a better standardization of the composition of the raw material, a greater process reproducibility, and a more reproducible product quality. While the use of milk protein concentrates may be a preferential tool to standardize fresh milk composition, the use of RM alone or of mixtures of fresh milk and RM may be a suitable practice to face off local and/or temporal shortage of milk, preserving the presence of cheeses on the market. Although much literature on the use of MP in dairy technology is available, to our knowledge, studies on its applicability for industrial, not stretched, soft cheeses are still scarce. The aim of this work was to evaluate the effect of 100% recombined milk (RM), mixed in different ratios (40 and 60%) with fresh milk, on the processing technology and quality of Crescenza, an Italian soft cheese. To prepare RM, a commercial skimmed MP, with quality comparable to a medium-heat and very close to a low-heat milk powder, was used. Cheeses were manufactured in a mini plant in laboratory scale, specially set up to simulate the cheesemaking process, from the continuous heat exchange system of pasteurization to the cheese storage. To assess the validity of cheese production on a laboratory scale, the properties of control cheese (obtained with fresh milk) were compared with those of the most consumed commercial Crescenza, taken from retail. Since no significant differences were found between commercial and control cheeses, and the latter were found to be in accordance with the compositional standards described for Crescenza [20], it was concluded that the use of a cheese model system was able to simulate the real conditions of cheesemaking. The advantage of using such a system consists of the possibility of simulating very different experimental conditions or changes in technology, thus obtaining, with acceptable approximation, useful indications on the processing of cheese in situations closer to industrial reality.

The replacement of fresh milk with 60–100% RM worsened the milk coagulation properties, and the products showed a moisture retention higher than control cheese, probably due to the presence of denatured whey proteins adhered to the casein micelles or the formation of large aggregates [22,23], which can entrap more water. This trend confirmed previous findings on Mozzarella [11] and could be related to the accentuated water-holding capacity of denatured whey proteins, leading to a softer coagulum [6]. The average moisture content of Crescenza-type cheeses produced with 100% RM was within the variability of the water content detected in Crescenza cheeses taken from retail. The moisture content was responsible for the actual yield of Crescenza-type cheeses made with 100% RM, which was significantly higher than control cheese, thus confirming previous findings on Cheddar cheese [24]. However, an excessively high moisture content may negatively impact cheese quality and shelf-life, especially in terms of proteolysis, textural softening, and product freshness [17,25]. With 40% RM, the differences between control and Crescenza-type cheeses tended to decrease.

Cheeses produced with any percentage of RM were of acceptable quality but, although they fell within the standard characteristics established for Crescenza cheese [20], they remarkably differed from the corresponding cheeses obtained from fresh milk in terms of sensory properties. Minor process modifications minimized the differences between cheeses with 40% RM and the control cheese. The addition of CPC to 40% RM allowed us to obtain Crescenza-type cheeses that were more similar to the control cheese in terms of coagulation parameters and syneresis. The employment of CPC leads to a greater aggregation of the micelles by increasing the inter-micellar Ca-P bridges, which is one of the most important factors to restore the renneting coagulation properties of a treated milk [26]. The use of CaCl_2_ is a cheaper solution to improve milk renneting properties. The addition of 0.05 g/L CaCl_2_ to 40% RM and a reduction of the grain size of the curd allowed us to obtain Crescenza-type cheeses with composition and sensory properties comparable to the control cheese. Amounts of CaCl_2_ higher than 0.05 g/L, which is one of the known causes of cheese bitterness [27,28], were not necessary in our trials. To effectively reduce the grain size of the curd, a specific cutting tool with a reduced distance between the knives was used.

## 5. Conclusions

This study showed the applicability of recombined skimmed milk (RM) in the manufacture of Crescenza-type cheese, although the success of this practice depends on various factors, including the technology, the quality of the MP to be used in cheese production, and the quantity of RM to replace fresh milk. To this regard, our study showed that 40% RM is a suitable amount to obtain Crescenza-type cheeses similar to controls, i.e., cheeses made with 100% fresh milk. It is essential to guarantee the suitability, in terms of renneting properties and absence of off flavors, of the MP to be reconstituted in fresh milk to obtain the RM. In this context, a medium-heat MP, which is more widespread and has a lower cost than a low-heat MP, was employed. The addition to 40% RM of technological adjuvants permitted by law (such as CPC or CaCl_2_), combined with the application of minimal processing changes, allowed us to obtain Crescenza-type cheeses with characteristics comparable to the controls. Economic feasibility assessments are necessary to accurately examine the pros and cons regarding the use of MP in the dairy industry.

## Figures and Tables

**Table 1:** Moisture, total fat, total proteins, ash, lactose, galactose, lactic acid, pH, and the fat to protein ratio (F/P) of Crescenza-type cheeses manufactured with 100% fresh milk (control) and four commercial cheeses (named cheese-1, cheese-2, cheese3, and cheese-4) from retail.

-	Moisture %	Fat %	Protein %	Ash %	Lactose %	Galactose %	Lactic Acid %	pH	F/P
Control	56.98	23.49	15.21	2.21	0.86	0.92	0.88	5.15	1.54
cheese-1	59.99	23.83	12.17	1.86	1.32	0.71	0.68	5.25	1.95
cheese-2	58.40	23.19	14.73	1.90	0.62	0.91	0.74	5.15	1.57
cheese-3	61.28	20.36	13.98	1.82	0.74	1.03	0.74	5.22	1.46
cheese-4	55.48	27.72	12.47	2.19	1.03	0.74	0.65	5.23	2.22

**Table 2:** Renneting properties of the recombined milk (RM) reconstituted at the same protein and fat content of fresh raw milk and used alone or with the addition of 0.1 g/L CaCl_2_ or 1 mL/L colloidal calcium phosphate (CPC). Raw and pasteurized liquid milk were used as controls.

Sample	r (min)	K20 (min)	A30 (mm)
Raw milk	10.77	4.00	43.61
s. d.	(0.37)	(0.32)	(2.42)
Pasteurized milk	11.56	4.04	43.58
s. d.	(0.32)	(0.40)	(3.69)
RM	16.6 *	8.38 *	30.31 *
s. d.	(0.73)	(0.97)	(3.13)
RM + CPC	9.04 *	6.04 *	31.68 *
s. d.	(0.38)	(0.82)	(2.99)
RM + CaCl_2_	11.61	9.29 *	30.32 *
s. d.	(0.44)	(1.08)	(2.06)

* significant differences (*p* < 0.05).

**Table 3:** Clotting time (CT) and fermentation time to pH 5.2 during processing. Actual (Ya), corrected (Ymafpam) cheese yield and gross composition of Crescenza-type cheeses produced with different concentrations of recombined milk (RM) and with 100% fresh milk (control cheese) were determined after 7 days of maturation.

-	CT	Time to Reach pH 5.2	Moisture	Fat	Protein	Ya	Ymafpam
-	(min)	(h)	(%)	(%)	(%)	(%)	(%)
RM 100%	16.97 *	4.66	61.27	19.50	14.30	18.54 *	17.06
s. d.	(1.77)	(0.66)	(1.86)	(1.24)	(0.72)	(0.70)	(0.71)
RM 60%	15.50 *	4.62	60.31 *	20.05 *	15.06	17.63	17.37
s. d.	(2.86)	(0.40)	(0.95)	(0.33)	(0.44)	(1.30)	(0.59)
RM 40%	13.72	4.74	59.86	20.60	14.84	18.05	17.68
s. d.	(0.71)	(1.21)	(1.88)	(1.21)	(0.84)	(1.65)	(0.40)
Control cheese	12.09	4.49	58.13	22.37	15.29	17.09	17.32
s. d.	(0.81)	(0.51)	(1.88)	(1.05)	(0.91)	(0.83)	(0.21)

* significant differences (*p* < 0.05).

**Table 4:** Technological parameters determined in the Crescenza-type cheeses produced with 40% of recombined milk (RM) added with 0.5 mL/L CPC or with 0.05 g/L CaCl_2_ followed by the cut change to reduce curd grain size; 100% fresh milk (control cheese). Clotting time, evolution of syneresis during processing, actual (Ya), and corrected (Ymafpam) cheese yields are reported.

-	Clotting Time	Syneresis (% *w*/*w*)					Ya	Ymafpam
-	(min)	Molding	1 Turning	2 Turning	3 Turning	Total	(%)	(%)
RM 40% + CPC	13.52	49.23	14.07	9.47	6.39	79.16	16.76	16.81
s. d.	(1.62)	(1.60)	(0.94)	(0.12)	(1.52)	(2.19)	(0.50)	(0.64)
RM 40% + CaCl_2_ + Cut change	14.29	51.82	15.60	9.17	3.97	80.56	16.59	16.86
s. d.	(0.95)	(3.17)	(1.26)	(0.49)	(0.69)	(1.87)	(1.10)	(0.29)
Controls	13.57	49.99	15.18	9.16	4.12	79.01	16.57	17.04
s. d.	(0.49)	(3.05)	(1.88)	(1.76)	(1.76)	(1.55)	(0.79)	(0.54)

s. d. = standard deviation. For each parameter, no significant differences were detected.

**Table 5:** Composition of the whey drained after manufacture and the Crescenza-type cheeses after maturation (7 days) using 40% recombined milk (RM), added with 0.5 mL/L of CPC or 0.05 g/L CaCl_2_ + cut change (see Figure 1). Control: cheeses made with 100% fresh milk.

-	Whey Drained (%)				Cheeses (%)						
-	Moisture	Fat	Protein	Lactose	Moisture	Fat	Protein	Ash	Lactose	Galactose	Lactic acid
RM 40% + CPC	92.23	0.31	0.94	5.23	56.73	23.80	15.11	2.33	1.01	0.83	0.75
s. d.	(0.05)	(0.03)	(0.01)	(0.09)	(0.74)	(0.66)	(0.22)	(0.30)	(0.08)	(0.12)	(0.13)
RM 40% + CaCl_2_ + cut change	92.22	0.34	0.92	5.24	57.21	23.04	15.46	2.49	1.00	0.80	0.71
s. d.	(0.15)	(0.03)	(0.02)	(0.08)	(0.83)	(1.49)	(0.61)	(0.26)	(0.07)	(0.06)	(0.04)
Control	92.40	0.27	0.95	5.12	57.01	23.09	15.55	2.40	0.90	0.81	0.75
s. d.	(0.11)	(0.04)	(0.04)	(0.08)	(1.50)	(1.78)	(0.57)	(0.28)	(0.06)	(0.05)	(0.06)

s. d. = standard deviation. For each parameter, no significant differences were detected.

**Table 6:** Sensory analysis obtained from the triangle tests comparing recombined Crescenza-type cheeses (RM) with the control cheese (C).

	Differences Detected (%)	Significance (*p* < 0.05)
**RM 100% vs. C**	67.12	***
**RM 60% vs. C**	72.06	**
**RM 40% vs. C**	57.53	**
**RM 40% + CPC vs. C**	58.44	**
**RM 40% + CaCl_2_ + Cut vs. C**	43.37	-

*** Significant differences detected three times out of three replicates. ** Significant differences detected two times out of three replicates. - No difference.
